# Computational Phenotyping of Two-Person Interactions Reveals Differential Neural Response to Depth-of-Thought

**DOI:** 10.1371/journal.pcbi.1002841

**Published:** 2012-12-27

**Authors:** Ting Xiang, Debajyoti Ray, Terry Lohrenz, Peter Dayan, P. Read Montague

**Affiliations:** 1 Department of Neuroscience, Baylor College of Medicine, Houston, Texas, United States of America; 2 Computation and Neural Systems, California Institute of Technology, Pasadena, California, United States of America; 3 Virginia Tech Carilion Research Institute and Department of Physics, Virginia Tech, Roanoke, Virginia, United States of America; 4 Gatsby Computational Neuroscience Unit, University College London, London, United Kingdom; 5 Wellcome Trust Centre for Neuroimaging, London, United Kingdom; Indiana University, United States of America

## Abstract

Reciprocating exchange with other humans requires individuals to infer the intentions of their partners. Despite the importance of this ability in healthy cognition and its impact in disease, the dimensions employed and computations involved in such inferences are not clear. We used a computational theory-of-mind model to classify styles of interaction in 195 pairs of subjects playing a multi-round economic exchange game. This classification produces an estimate of a subject's depth-of-thought in the game (low, medium, high), a parameter that governs the richness of the models they build of their partner. Subjects in each category showed distinct neural correlates of learning signals associated with different depths-of-thought. The model also detected differences in depth-of-thought between two groups of healthy subjects: one playing patients with psychiatric disease and the other playing healthy controls. The neural response categories identified by this computational characterization of theory-of-mind may yield objective biomarkers useful in the identification and characterization of pathologies that perturb the capacity to model and interact with other humans.

## Author Summary

Human social interactions are extraordinarily rich and complex. The ability to infer the intentions of others is essential for successful social interactions. Although most of our inferences about others are silent and subtle, traces of their effects can be found in the behavior we exhibit in various tasks, notably repeated economic exchange games. In this study, we use a computational model that uses an explicit form of other-modeling to classify styles of play in a large cohort of subjects engaging in such a game. We classify players according to their depth of recursive reasoning (depth-of-thought), finding three groups whose performance throughout the task differed according to several measures. Neuroimaging results based on the model classification show a differential neural response to depth-of-thought. The model also detected differences in depth-of-thought between two groups of healthy subjects: one playing patients with psychiatric disease and the other playing healthy controls. These results demonstrate the power of a quantitative approach to examining behavioral heterogeneity during social exchange, and may provide useful biomarkers to characterize mental disorders when the capacity to make inferences about others is impaired.

## Introduction

Many of the inferences that we make about others, or about their models of us, are silent and subtle [1], [2]. One route for understanding the neural basis of such inferences comes from building computational models of social exchange that quantify their nature and evolution over the course of interactions. Recent behavioral and neuroimaging work in this area has employed interactive economic games that required subjects to model their partners' strategies [3]–[6]. This work focused on relatively small cohorts of subjects, or on subjects knowingly playing a computer partner. Therefore, questions about individual differences in styles of play, and whether or not the partner was treated by the brain like a human partner remain largely open (but see 6).

**Figure 1:**
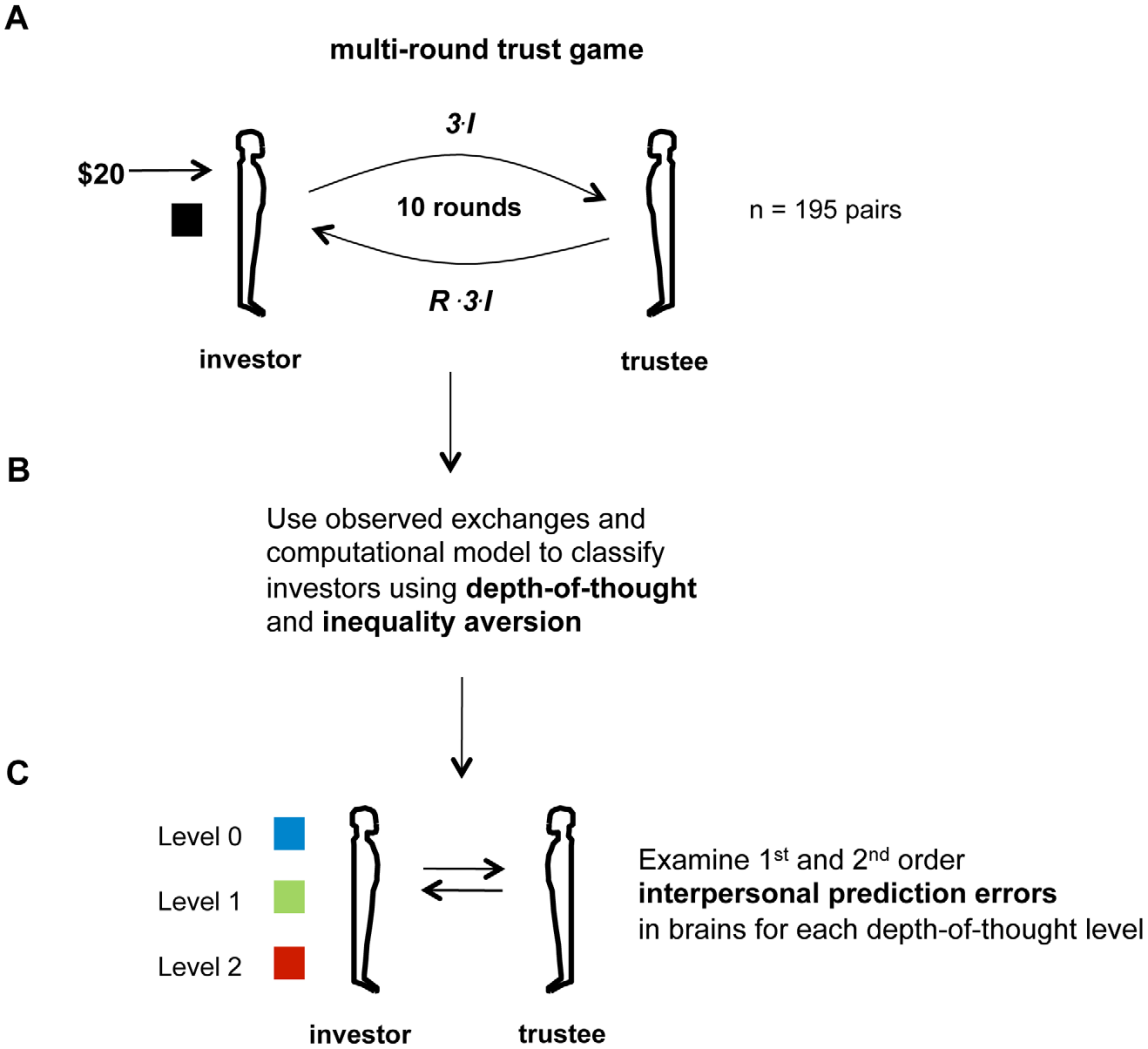
Classification of investors. A) One player (“investor”) is endowed with $20 at the beginning of each round. The investor chooses any fraction *I* of the $20 to send to the other player (“trustee”). The investment is tripled to 3I en-route to the trustee. The trustee chooses a fraction *R* of the tripled amount (*3I*) to repay. Subjects play the same partner for ten consecutive rounds. B) Using the observed exchanges between the players, investors are classified according to their estimated inequality aversion and their depth-of-thought (strategic level) in the game (see main text for a description of the generative model). All 195 pairs included in this classification; this included 55 pairs where the trustee was diagnosed with Borderline Personality Disorder. C) First and second order interpersonal prediction errors are sought in the investors' brain responses separately for each depth-of-though category. The 1^st^ order interpersonal prediction error is taken as the difference between actual repayment ratio *R* and expected amount due to the investor's model of the trustee's repayment. The 2^nd^ order prediction error is taken as the difference between the investment ratio *I* and the investor's model of the trustee's model of what the investor will send; hence, the term second order error.

Figure 1 outlines the strategy of the approach. We used a multi-round reciprocation game (the multi-round trust game, Figure 1A), classifying the play of a large (n = 195) number of pairs of players (dyads) [7]–[9] via a computational realization of the models of each other that they build [10]. This classification used the observed patterns of monetary exchange to infer two parameters important for all such exchanges: (1) the sensitivity of a subject to deviations from fair splits of money across the two players (called inequality aversion) [11]; and (2) the subject's depth-of-thought or cognitive level in the game, that is, the depth to which they modeled their interaction with their partner [12]. After classification along these two dimensions, we sought neural correlates of learning signals (interpersonal error signals) inferred by the model that are important for playing the game successfully (Figure 1B and 1C). We describe the model below.

A player's type is represented by her degree of inequality aversion. Players value immediate payoffs, but to a lesser degree if the split of money between them is inequitable [13]: 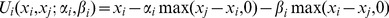
(1a)where 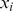 is the money obtained by player *i* and 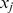 is the amount obtained by player *j*. Two sorts of inequity are potentially important: envy (partner *j* gets more than subject *i* ; 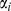 in eqn 1a) and guilt (subject *i* gets more than partner *j*; 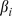 in eqn 1a). The envy and guilt parameters comprise what we consider as the type of a player. Empirically, the majority of investors invest more than half of the endowment and the modal behavior of trustees is to split the sum of money evenly. Hence, the influence of “envy” on subjects' choices was minimal. For simplicity, we assume 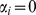 and consider only “guilt” - the aversion to inequity favorable to the subject – as the way to type a player. Therefore, player *i*'s type is fully described by 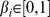, the “guilt” parameter. The utility function becomes: 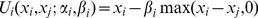
(1b)


The second important feature of the model is depth-of-thought in the game [12], which derives from the estimates that each player maintains about the type of their partner. To maximize long-run utility, a player must estimate this type, and update the estimate when observing their partner's actions. Of course, I may estimate your type, your estimate of my type, your estimate of my estimate of your type, and so forth [14]. Thus we define deeper thinkers in the game as those who use more sophisticated simulations of play of this sort to update these estimates.

A range of behavioral data suggests one strong constraint on how subjects model their partners, that is, they assume that their partners play one level less sophisticated than themselves [15]. We assume that all players plan ahead and choose actions that have beneficial consequences, but differ in how they interpret the signals from their partners to update their beliefs, and how they expect their partners perceive them through their actions. To estimate one's partner's type, a level 0 subject does not simulate his partner's play, but assumes his partner is level 0 i.e. also has a naïve model of them. A level 1 subject assumes his partner is a level 0 player and simulates how a level 0 partner makes choices. A level 2 subject assumes his partner is level 1 and simulates how a level 1 player interacts throughout the game. This kind of recursion lies at the heart of mentalizing (simulating) other autonomous agents who concurrently generate models of us – it also lies at the heart of many models of predator-prey interactions [16].

### The computational model of behavior – simulating interactions with one's partner

Here we write the model for how player 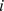 forms an estimate of optimal play at each round *t* by calculating the values 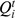 of their possible actions 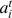. The actions are the amounts to invest or to return. The 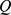 values are the expected summed utilities over the next two rounds (as a simplification, players are assumed to look at the current round and the round after). The utility for player *i* depends on the actions of player *j*, which in turn depends on the type of player *j*, and the reasoning that player *j* does about player *i*. Player *i* does not know player *j*'s type, but can learn about it from the history of their interactions, which, up to round *t*, is 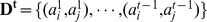. Formally, player *i* maintains beliefs 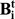, in the form of a probability distribution over the type of player *j*, and computes expected utilities by averaging over these beliefs. Bayes theorem is used to update the beliefs based on evidence.

The 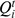 value on round t is a sum of two expectations: 
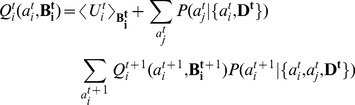
(2)


The first is the utility of the exchange on that round. This is
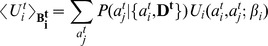
where, for convenience, we write 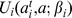 as a function of the possible actions *a* of player *j* rather than the money this player earns. The second term in equation (2) concerns the value of future 2 rounds in the exchange (except in the last round, where this term is 0). This is thus an average over 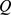 values 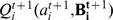 on round *t+1*, where the new beliefs 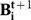 take account of the action 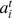 being considered by player *i*, and all the possible actions 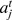 of player *j*. Equation (2) is a form of Bellman evaluation equation.

The players can calculate the 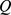 values, including updating the beliefs, by simulating the course of play with their partners. This simulation is a central feature of the model with players adopting higher levels of depth-of-thought requiring more simulation (see belief updates in Supporting Information).

## Results

### Classification of interpersonal interaction

The model described above constitutes a full generative account of a subject playing the multi-round trust game, and incorporates several key cognitive mechanisms engaged by such a staged interpersonal interaction. Player *i* is characterized by their private type 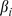, their depth-of-thought level 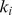, and the prior beliefs 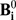. Player *j* is characterized in just the same way. We estimated the parameters of both players in each dyad by maximizing the log likelihood of their choices over the 10 rounds of the game. The averaged maximal log likelihood of all 195 investors was −11.92±0.27. In our model, we assume that players take one of five possible actions. If all the five possible actions were chosen with equal probability, the log likelihood would take the value 
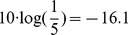
. Our computational theory-of-mind model fitted the behavior significantly better than assuming that players act randomly (one-sample test, *P* = 1.51×10^−35^). For the purposes of comparison, we also built a reinforcement learning (RL) model incorporating inequality aversion (details in Supporting Information). We found that the RL model performed poorly; when we optimized the learning rate in the model, the optimum was degenerate in the sense that no learning occurred, and all actions were selected with equal probability (random choices).

**Figure 2:**
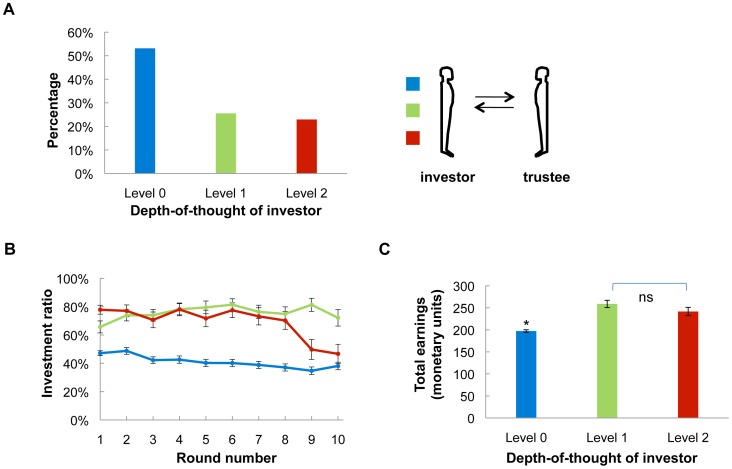
Investor depth-of-thought classification separates distinct behavioral trajectories through the game. A) The distribution of depth-of-thought levels in all 195 investors. About half of the investors are classified as having depth-of-thought level 0. The remaining half is almost equally divided into having depth-of-thought level 1 and 2. B) Investment ratios by rounds from all three levels of depth-of-thought investors, level 0 (n = 102), level 1 (n = 49), level 2 (n = 44). C) Total monetary points earned at the end of the game in all three levels of investors. Both level 1 and level 2 investors made significantly more points than level 0 investors (Tukey HSD test, *P*<10^−6^ and *P*<10^−5^, respectively). No significant difference in total earnings was found between level 1 and level 2 investors (*P*>0.1). Error bars represent standard errors (SE).

Figure 2A shows the frequency histogram of depth-of-thought classification achieved by inverting the generative model described above. About half of all the 195 investors are classified as strategic level 0. The remaining investors are almost equally divided into level 1 and level 2 players. There are significant dynamic behavioral features that correlate with the depth-of-thought levels that we estimate using our model. The style of play across rounds of the game is different and correlates well with the intuition that players with higher depths-of-thought are sensitive to richer features of the game than those possessing lower levels. In Figure 2B, of all 195 investors, levels 1 and 2 start the game with high offers and maintain throughout the game, except that the highest depth-of-thought players decrease their offers towards the end of the game (which is strategic). Moreover, level 0 investors open low and stay low throughout the game, a strategy that tends to break cooperation with the trustee. Lastly, level 1 and 2 players make significantly more money overall than level 0 players (Figure 2C).

### Neural representations

According to the generative model, players make predictions about the likely course of events through the game. These predictions lead naturally to prediction errors, which can be used to generate control signals to guide choices. In games against nature, prediction errors associated with rewarding outcomes have frequently been observed in the BOLD signal measured in striatal regions [17]–[19]. Games against other players offer much richer possibilities for neural responses since players have a range of interpersonal signals that they can model (e.g. Figure 1C). We here focus on the investor side of the interaction because this role has proved to be particularly sensitive for classifying styles of play in prior work [20].

Two types of interpersonal prediction errors emerge naturally in the reciprocating interactions of the multi-round trust game. The first order prediction error in the investor is a comparison between the investor's current model of what the trustee will return and the amount actually returned. This error is computed at the time that the repayment from the trustee is revealed to the investor. This error requires information sent back from the trustee.

By contrast, the second order prediction error in the investor requires a comparison between the investor's offer and the investor's internal model of what the trustee expects from the investor, that is, information that is exclusively internal to the investor. This information is available to the investor before any immediate feedback from the trustee, and is potentially available during the entire epoch, starting from the time of the cue and up until the time when the actual investment is made. In this paper, we choose the time the investor submits as a natural trigger for this signal, but with the understanding that it might have been computed and thus available earlier.

Thus, the first order error can be evaluated at the time the repayment from the trustee is revealed. In a similar spirit, the second order error is defined at the time the investor's offer is submitted since it is at this time that the investor brain can compare their actual offer to their (internal) model of what the trustee expects.

Our hypothesis for the first order inter-personal prediction error was that players classified as level 0 would display a large response to this error, while the higher levels would not, since this signal is not a critical component of the high level players' planning.

**Figure 3:**
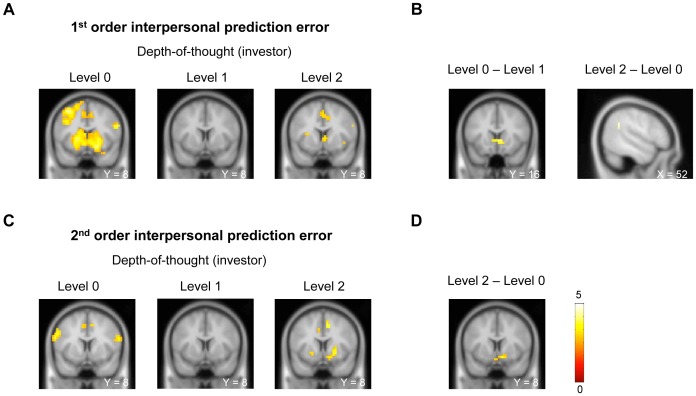
Inter-personal prediction errors: differential neural response as a function of investor depth-of-thought. A) Contrast analysis between rounds with high (>60%) and low (≤40%) 1^st^ order interpersonal prediction errors when repayments were revealed. Level 0 investors (n = 102) had robust activations in bilateral striatal regions (whole-brain FDR corrected at *P*<0.05; peak MNI coordinates: caudate (8, 12, 0), t = 4.49; putamen (24, 4, 0),t = 4.02). These striatal activations were not observed in investors with level 1 (n = 49) or level 2 (n = 44) depth-of-thought. B) Group contrast analysis on the 1^st^ order interpersonal prediction errors. Left, level 0 investors had higher caudate activation than level 1 investors (*P*<0.001, uncorrected; peak MNI coordinates: (4, 16, 0), t = 4.04, FWE corrected at *P*<0.05 with small volume correction applying the anatomical mask of bilateral caudate). Right, level 2 investors had higher right temporal-parietal junction (TPJ) activation than level 0 investors associated with the 1^st^ order interpersonal prediction errors (whole-brain FDR corrected at *P*<0.05; peak MNI coordinates: (52, −48, 28), t = 4.70, 7 voxels). C) Contrast analysis between rounds with high (>60%) and low (≤40%) 2^nd^ order interpersonal prediction errors when investments were submitted. Level 2 investors had significant activations in bilateral putamen (whole-brain FDR corrected at *P*<0.05; peak MNI coordinates: putamen (24, 8, −4), t = 3.79). We did not observe any striatal activations in level 0 and level 1 investors for the 2^nd^ order prediction errors. D) Group contrast analysis on the 2^nd^ order interpersonal prediction errors. Level 2 investors had higher ventral striatal activation than level 0 investors when computing the 2^nd^ order interpersonal prediction errors (*P*<0.005 uncorrected; peak MNI coordinates (12, 8, −12), t = 3.41, FWE corrected at *P*<0.05 with small volume correction applying the anatomical mask of bilateral caudate). Color bars display t scores.

We divided the first order interpersonal prediction error of all 195 healthy investors classified within a certain cognitive level into quintiles, performed separate GLM analysis at individual rounds, and then generated contrasts between rounds with high 1^st^ order prediction errors (>60%) and rounds with low 1^st^ order prediction errors (**≤**40%) on the beta images of the events of interest. The contrast analysis at the revelation of the trustee's repayment showed that level 0 investors (n = 102) had robust activations in bilateral striatal regions (Figure 3A, whole-brain FDR corrected at *P*<0.05; peak MNI coordinates: right caudate (8, 12, 0), t = 4.49, 57 voxels; left caudate (−12, 12, 4), t = 3.74, 73 voxels; right putamen (24, 4, 0),t = 4.02, 88 voxels; left putamen (−24, 4, 4), t = 4.10, 72 voxels). These striatal activations were not observed in investors with level 1 (n = 49) or level 2 (n = 44) depth-of-thought. We also performed a direct comparison among investors with different depth-of-thought levels on the 1^st^ order interpersonal prediction errors using ANOVA. The group contrast results showed that the level 0 investors had higher caudate activation than level 1 investors (Figure 3B left, *P*<0.001, uncorrected; peak MNI coordinates: (4, 16, 0), t = 4.04, FWE corrected at *P*<0.05 with small volume correction applying the anatomical mask of bilateral caudate). We also found that level 2 investors had higher right temporal-parietal junction (TPJ) activation than level 0 investors associated with the 1^st^ order interpersonal prediction errors (Figure 3B right, whole-brain FDR corrected at *P*<0.05; peak MNI coordinates: (52, −48, 28), t = 4.70, 7 voxels).

Our hypothesis for the second order inter-personal prediction error was that players classified as level 0 would display no response to this higher order interpersonal error (since their model of the other's model of themselves is impoverished), whereas players classified as higher level would.

We divided the second order inter-personal prediction error of all 195 healthy investors classified within a certain cognitive level into quintiles, performed separate GLM analysis at individual rounds, and then generated contrasts between rounds with high 2^nd^ order prediction errors (>60%) and rounds with low 2^nd^ order prediction errors (**≤**40%) on the beta images of the events of interest. The contrast at the submission of the investor's decisions revealed that level 2 investors had significant activations in bilateral putamen (Figure 3C, whole-brain FDR corrected at *P*<0.05; peak MNI coordinates: right putamen (24, 8, −4), t = 3.79, 23 voxels; left putamen (−20, 8, −4), t = 3.11, 7 voxels). We did not observe any striatal activations in level 0 and level 1 investors for the 2^nd^ order prediction errors. We also performed an ANOVA analysis on the three depth-of-thought levels of investors. The group contrast analysis found that level 2 investors had higher ventral striatal activation than level 0 investors when computing the 2^nd^ order interpersonal prediction errors (Figure 3D, *P*<0.005 uncorrected; peak MNI coordinates (12, 8, −12), t = 3.41, FWE corrected at *P*<0.05 with small volume correction applying the anatomical mask of bilateral caudate).

**Figure 4:**
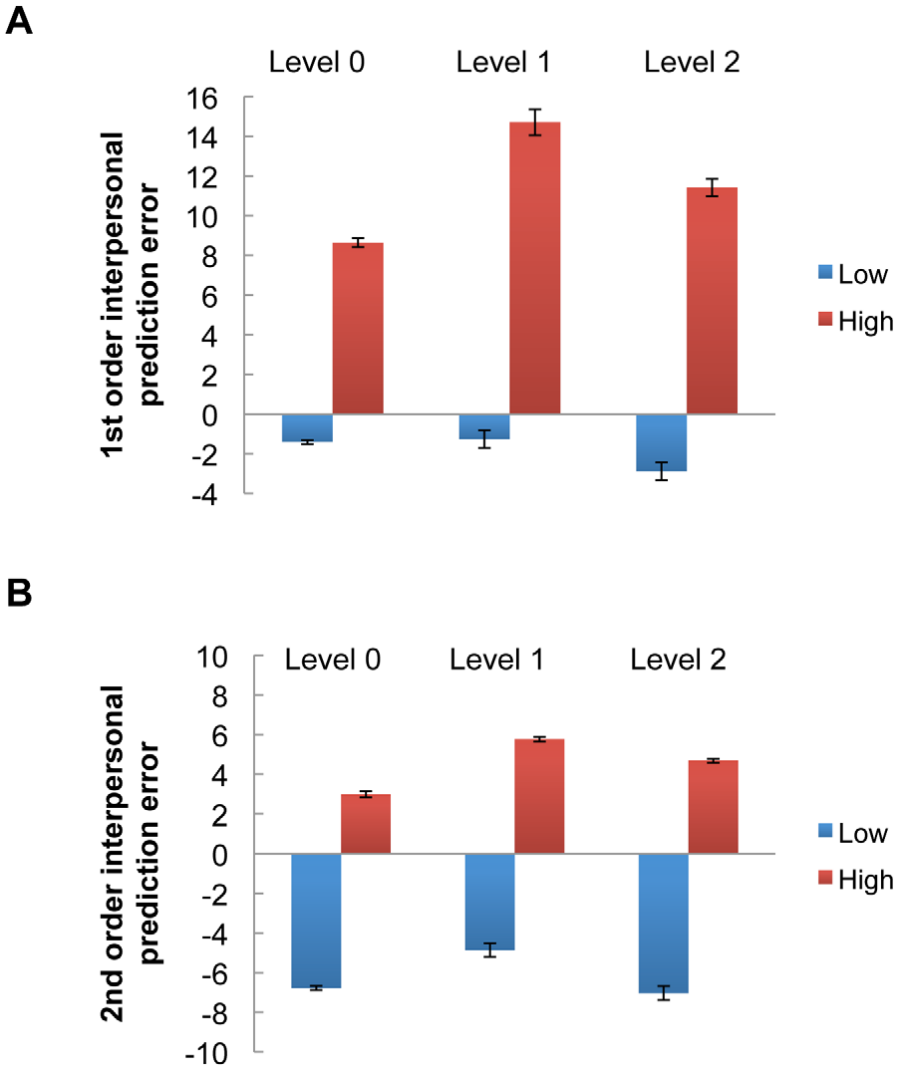
Magnitude of interpersonal prediction errors as a function of estimated depth-of-thought for investors. Average 1st order A) and 2nd order B) inter-personal prediction errors: low (bottom two quintiles), high (top two quintiles). The differences between the high and low 1^st^ order interpersonal prediction errors were as follows: level 0 investors (mean = 10.05, SE = 0.38), level 1 investors (mean = 15.97, SE = 0.55), level 2 investors (mean = 14.30, SE = 0.58). The differences between the high and low 2^nd^ order interpersonal prediction errors were: level 0 investors (mean = 9.76, SE = 0.22), level 1 investors (mean = 10.62, SE = 0.31), level 2 investors (mean = 11.72, SE = 0.33).

It is possible that when grouping the rounds according to the high or low quintiles of prediction errors, some subjects might be exclusively included in the high group, or in the low group. This raised the concern that the contrast results above might be biased by those distinct subjects. We therefore counted the number of subjects only present in the high group, or in the low group for the 1^st^ and 2^nd^ interpersonal prediction errors, respectively. We showed that the vast majority of subjects made contributions to all quintiles of prediction errors, with only an extremely small number of subjects contributing to just the high or low quintiles (Table S1). We also plotted the magnitudes of the interpersonal prediction errors divided into high or low quintiles across the depth-of-thought levels. We did this to rule out the possibility that a few subjects were dominating the observed results. The differences between the high and low quintiles were comparable across all the three levels of investors for both the 1^st^ and 2^nd^ order interpersonal prediction errors (Figure 4). Thus, the differential neural activations to the prediction errors observed here cannot be attributed to the differences in the magnitudes of prediction errors per se.

### Biosensor manipulation: Trustee ‘types’ induce depth-of-thought distributions in healthy investors

**Figure 5:**
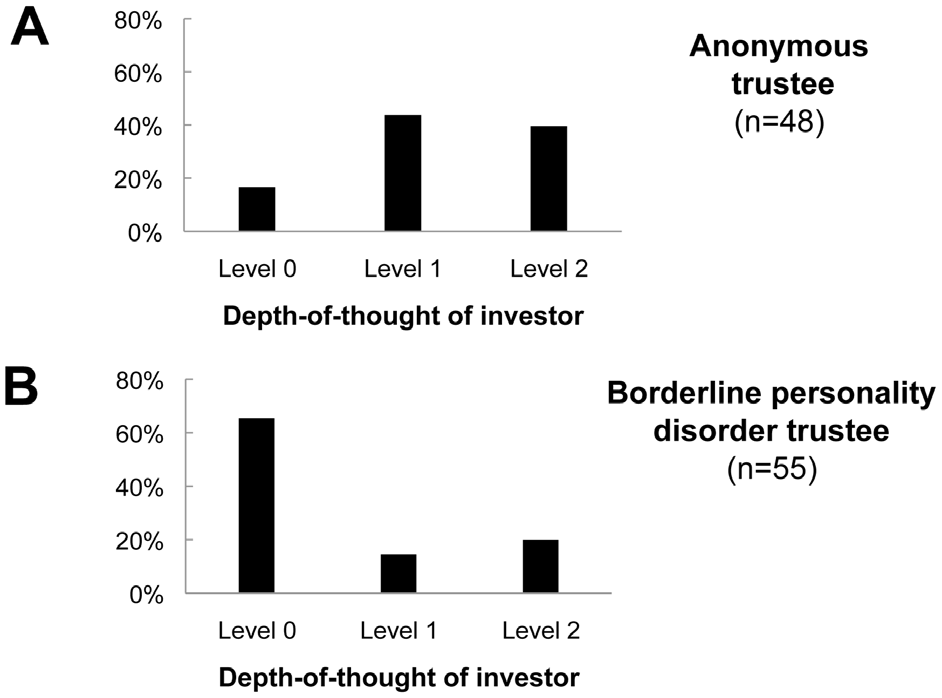
Distribution of depth-of-thought in investors as a function of trustee group. A) Anonymous trustees (n = 48) remain anonymous to their investor partner for the entire game (and visa versa). B) Borderline personality disorder trustees were identified through an extensive set of formal interview procedures (see King-Casas et al., 2008). On Fisher's exact test, the borderline personality disorder-induced investor depth-of-though distribution was significantly different from investors playing anonymous trustees (panel A; p = 1.68×10^−6^).

Earlier work [9] found that trustees diagnosed with Borderline Personality Disorder (BPD) played uncooperatively to an extent that they could not maintain the cooperation of their partner investor. In that work, the impact of the trustee behavior was ‘read out’ through the willingness of the investor to sustain high offer levels throughout the rounds of the game. Figure 5 shows two distributions of estimated investor depth-of-thought levels as a function of distinct trustee types.

Panel A shows the distribution when healthy investors play anonymous healthy trustees (n = 48 pairs). In this exchange, healthy subjects never meet their partner before the game and do not see or meet them after the game. They arrive at the lab and are randomly assigned roles in separate rooms. Panel B shows the distribution when healthy investors play subjects diagnosed with borderline personality disorder. There is a more dramatic shift toward lower depth-of-thought levels despite the fact that these subjects play the healthy investor anonymously. The distributions in panels A and B are statistically different (see legend Figure 5). We also recruited 38 trustee matched for lower socio-economic scale (SES) as a SES match for the Borderline personality disorder trustees. These trustees also played anonymously and induced a similar lower depth-of-mind distribution in the investors (Figure S2) suggesting that lower SES may be one source of influence for the incapacity of the Borderline subjects to sustain cooperation with their investor partners.

## Discussion

In this paper, we used a Bayesian computational model that involves an explicit representation of theory of mind to classify a large number of subjects playing an economic exchange game. We used the model to assess their level of depth-of-thought. Our classification produces three levels of players whose behaviour correlates with important measures of performance through the task. Neuroimaging results based on the model classification showed a differential response to depth-of-thought. Additionally we found a significant difference for investor depth-of-thought distributions when comparing play with healthy trustees to play with subjects diagnosed with borderline personality disorder (BPD), a disorder known to disrupt inter-personal interactions. BPD subjects are characterized by their unstable relationships, and when they have played this game, they have tended to break cooperation. Indeed, it has been shown that, for this group, the anterior insula failed to sense the opponent's low offers [8].

The striatum has long been shown to encode reward prediction error signals in both passive and instrumental conditioning tasks [17], [21]–[23]. Recently striatal activation has also been observed in social learning tasks [24] and tasks requiring mentalizing a partner's intention [3]. Here we found that striatum activity correlated with two types of interpersonal prediction errors evoked in a repeated social exchange game, and that these signals were modulated by players' depth-of-thought levels. Level 0 players, but not level 2 players, had robust activations in the striatum to high 1^st^ order interpersonal prediction errors suggesting the naïve players were particularly sensitive to opponent's actions and mainly used this type of errors to adjust their own action policy. However, the striatum in level 2 players responded only to the 2^nd^ order interpersonal errors suggesting that these relatively sophisticated players discounted the direct influence of opponent's actions and rather put more emphasis on simulating and manipulating opponent's beliefs and actions. Other imaging experiments requiring subjects to model others' intentions have also reported activations in frontoparietal regions [3], [5], [24]. It is not clear why frontoparietal regions were not observed in our paradigm. However, there is a clear path from known error signaling in the striatum to our observations here of 2nd order inter-personal prediction errors, since a 2^nd^ order prediction error can be seen as a direct proxy for future returns to the investor. In this reciprocation game, we have previously reported that deviations from neutral reciprocity or tit-for-tat behavior cause players to change their behavior [7], [9]. Therefore, an investment that deviates positively from what the trustee expects (based on their model of the investor) should generate a positive error signal in the trustee's brain, which would itself lead to the investor expecting an increased return. Under this interpretation, the signal is exactly analogous to the range of prediction error signals that show up encoded in BOLD responses in the striatum. These neural results are congruent with our behavioral observations. The most sophisticated level 2 investors invested high at the beginning to cultivate trust and promote cooperation with their partners. But towards the end of the exchange, they responded to the horizon of the game and risked less money, reflecting their manipulative maneuver in the beginning. Furthermore, we found that the sophisticated level 2 investors had higher activations in the right TPJ in response to the 1^st^ and 2^nd^ order interpersonal prediction errors than the naïve level 0 investors. Right TPJ has been demonstrated to play a critical role in belief reasoning tasks involving “theory of mind” [25], [26]. Right TPJ has also been found to be specifically modulated in people with higher strategic levels [27]. Furthermore the coordinates of the peak voxel of this activation place it in a recently designated posterior region of the TPJ (TPJp) that is well-connected to “areas identified with social cognition” [28]. The TPJ activation and its specific location within TPJ is consistent with the idea that level 2 investors build more sophisticated models of their opponents.

Computational accounts developed in the framework of Markov Decision Processes (MDP), and in particular reinforcement learning models [29], have been successful in representing behavior and illuminating neural substrates in situations where agents interact with nature, and in which the environmental states are fully observable. Such models have furthered our understanding of the role of dopamine and related neural structures in reward learning and decision-making [30], [31]. However, those models are limited in the typical social situations where agents interact and effectively create an ever-changing, adapting landscape, which are plausibly a *raison d'etre* for sophisticated cognition. Recently, some progress has been made in establishing model-based approaches to social interaction [3], [4], [32], [33]. Our approach makes a commitment to an explicit, generative model of higher-order thinking about other social actors, some aspects of which are in common with the recent work by Yoshida et al. (who also use their models to compare autistic and healthy subjects) [4]–[6]. The space of such models is vast, and explicit choices must be made at many steps [4], [10]. Nonetheless, our model is able to capture striking heterogeneity in the behavior which we are then able to connect to differences in neural activity. Further developments of this approach also incorporating genetic data promise to help uncover the genetic underpinnings of social heterogeneity.

## Materials and Methods

### Ethics statement

Informed consent was obtained for all research involving human participants, and all clinical investigation was conducted according to the principles expressed in the Declaration of Helsinki. All procedures were approved by the Institutional Review Board of the Baylor College of Medicine.

### Subject characteristics

Data from four groups, total 195 pairs of subjects (18–64 yrs) who played the trust game previously [5]–[8] were examined, including an Impersonal group (48 pairs), a Personal group (54 pairs), a BPD group (55 pairs), and a BPD control group (38 pairs). Subject pairs from the Impersonal, BPD, and BPD control groups never met each other throughout the experiment. Subject pairs in the Personal group were introduced to each other before playing the task. Trustees in the BPD group were diagnosed with borderline personality disorder (BPD), and were matched to trustees in the BPD control group on socioeconomic status (SES). In addition, investors in the BPD and BPD control groups were recruited with socioeconomic status matched to trustees. Investors in the Impersonal groups were students from Caltech and Baylor College of Medicine.

### Image acquisition and preprocessing

All scans were carried out on 3.0 Tesla Siemens Allegra scanners. High-resolution T1-weighted scans (1.0 mm×1.0 mm×1.0 mm) were acquired using an MP-RAGE sequence (Siemens). Subjects then played the iterated trust game for 10 rounds while undergoing whole-brain functional imaging. The detailed settings for the functional run were as follows: echo-planar imaging, gradient recalled echo; repetition time (TR) = 2000 ms; echo time (TE) = 40 ms; flip angle = 90°; 64×64 matrix, 26 4-mm axial slices angled parallel to the anteroposterior commissural line, yielding functional 3.3 mm×3.3 mm×4.0 mm voxels.

Images were analyzed using SPM2 (http://www.fil.ion.ucl.ac.uk/spm/software/spm2/). Slice timing correction was first applied to temporally align all the images. Motion correction to the first functional image was performed using a 6-parameter rigid-body transformation. The average of the motion-corrected images was co-registered to each subject's structural images using a 12-parameter affine transformation. Images were subsequently spatially normalized to the Montreal Neurological Institute (MNI) template by applying a 12-parameter affine transformation, followed by nonlinear warping using standard basis functions. Finally, images were smoothed with an 8 mm isotropic Gaussian kernel and then high-pass filtered (128 s width) in the temporal domain.

### General Linear Model (GLM) analysis

Separate general linear models were specified for individual rounds of each subject (6). All visual stimuli, motor responses and motion parameters were entered as separate regressors that were constructed by convolving each event onset with a canonical hemodynamic response function in SPM2. Beta maps were estimated for regressors of interest. The SPM images shown in Figure 3 was generated as follows: both the first order and second order interpersonal prediction errors of subjects classified with the same depth-of-thought were divided into quintiles. For the 1^st^ order interpersonal prediction errors, beta images associated with the event when the repayments were revealed were sorted according to the prediction error quintiles. Contrast analysis between the beta images from top two quintiles (>60%) and images from the bottom two quintiles (**≤**40%) were performed. Similarly, contrasts for the 2^nd^ interpersonal prediction errors were generated from beta images associated with the event when the investments were submitted.

### Computational theory-of-mind model

See Text S1 for detailed descriptions. We also include a reinforcement learning model in Text S1 for comparison.

## Supporting Information

Figure S1 **Depth-of-thought distribution for investors playing trustee with lower SES.** Trustee group was matched to the SES of the identified BPD trustees, which tended to be lower than the average healthy trustee. In reciprocation games (including the multi-round trust game), it is known that lower SES correlates with lower offers and increased difficulty of sustaining cooperation. This investor depth-of-thought distribution suggests that reduced SES that can attend BPD may be one of the causative factors in their style of play; however, these data are simply consistent with that hypothesis and do not show causality. The lower SES trustees induce a depth-of-thought distribution that is significantly different from investors playing anonymous healthy trustees using Fisher's exact test (p = 1.99×10^−8^).
(TIF)

Figure S2 **Depth-of-thought distribution for investors playing healthy trustees non-anonymously.** Healthy trustees meet their investor partner at the beginning of the game and are paid in front of their partner at the end of the game. These subjects are not known to one another at the start of the game and are randomly assigned the role of trustee or investor. This depth-of-thought distribution is not statistically different from the distribution in figure S2 (Fisher's exact test p = 0.032).
(TIF)

Table S1 **Did a small number of subjects drive differences in the quintiles of inter-personal prediction errors?** The number of distinct subjects in low (bottom two quintiles only) and high (upper too quintiles only) 1^st^ and 2^nd^ order prediction errors, the total subjects in each category, and the percentage. Extremely few subjects were presented in the low or high categories only. The majority of investors made contributions to all the quintiles for both the 1^st^ and 2^nd^ order interpersonal errors, regardless of their depth-of-thought levels.
(TIF)

Table S2
**Parameters for reinforcement learning models.** Estimated parameters *k* and *b* for different learning rates 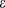 for reinforcement learning model.(TIF)

Table S3 **Model fit comparison.** Comparison of average negative log-likelihoods for reinforcement learning models using the estimated parameters, and the computational theory of mind model.
(TIF)

Table S4 **Joint classification table.** Joint Investor/Trustee depth-of-thought classification frequency table. Chi-Square test gives *p* = 6.4e-05.
(TIF)

Text S1 **Supplementary model information.**
(DOC)
