# Supplementary figures and images for: Computational Phenotyping of Two-Person Interactions Reveals Differential Neural Response to Depth-of-Thought
Source: PLoS Comput Biol. 2012 Dec 27;8(12):e1002841. doi: 10.1371/journal.pcbi.1002841 (PMC3531325; doi:10.1371/journal.pcbi.1002841)

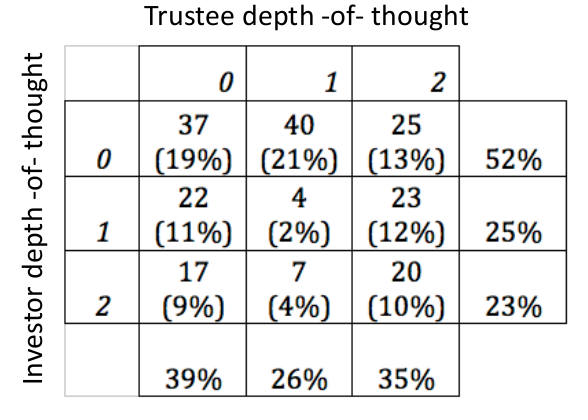

Supplement: Table S4 — Joint classification table. Joint Investor/Trustee depth-of-thought classification frequency table. Chi-Square test gives p = 6.4e-05. (TIF) [file pcbi.1002841.s006.tif]
