# Supplementary material for: Computational Phenotyping of Two-Person Interactions Reveals Differential Neural Response to Depth-of-Thought
Source: PLoS Comput Biol. 2012 Dec 27;8(12):e1002841. doi: 10.1371/journal.pcbi.1002841 (PMC3531325; doi:10.1371/journal.pcbi.1002841)
Supplement: Text S1 — Supplementary model information. (DOC) [file pcbi.1002841.s007.doc]

**Supporting Information**

**Computational theory-of-mind model**

***Player types defined by Fehr-Schmidt inequity aversion.*** As stated in the main text, a player’ type is represented by her degree of inequality aversion. Player *i* values immediate payoffs using the Fehr-Schmidt (1999) utility function (eqn 1):

$$U_{i}(x_{i},x_{j};\alpha_{i},\beta_{i})=x_{i}-\alpha_{i}\max(x_{j}-x_{i},0)-\beta_{i}\max(x_{i}-x_{j},0)\text{ }(1a)$$

where $x_{i}$ is the money obtained by player *i* and $x_{j}$ is the amount obtained by player *j*. Two sorts of inequity are important: envy (partner *j* gets more than subject *i* ; $\alpha_{i}$ in eqn 1a) and guilt (subject *i* gets more than partner *j*; $\beta_{i}$ in eqn 1a). The envy and guilt parameters comprise what we consider as the type of a player. Empirically, the majority of investors invest more than half of the endowment and the modal behavior of trustees is to split the sum of money evenly. Hence, the influence of “envy” on subjects’ choices was minimal. For simplicity, we assume $\alpha_{i}=0$ and consider only “guilt” - the aversion to inequity favorable to the subject – as the way to type a player. The utility function becomes:

$$U_{i}(x_{i},x_{j};\beta_{i})=x_{i}-\beta_{i}\max(x_{i}-x_{j},0)\text{ }(1b)$$

Therefore, player *i*’s type is fully described by $\beta_{i}\in[0,1]$, the “guilt” parameter. A player only knows her own type but not her opponent’s type. At any stage of the game, she maintains beliefs about the possible type of her opponent. Moreover, these beliefs are not restricted to first order beliefs about a partner’s type, but also include beliefs about the partner’s belief about their type and so on. Such situation is similar to a Partially Observable Markov Decision Process (POMDP) (32) or interactive POMDP (I-POMDP) (33). Here, we extend the POMDP or I-POMDP to a Bayesian game setting.

***Utility in the trust game.*** At round *t* of the ten-round trust game, the investor (with type $\beta_{i}$) starting with 20 points decides to send points $a_{i}^{t}$ to the trustee. The amount is then tripled, and the trustee (with type $\beta_{j}$) repays points $a_{j}^{t}$ back. The resulting payoffs of each player at the end of round t are:

Investor:

Trustee: $x_{j}^{t}(a_{i}^{t},a_{j}^{t})=3a_{i}^{t}-a_{j}^{t}$

The immediate utility for each player becomes:

Investor: $U_{i}^{t}(a_{i}^{t},a_{j}^{t};\beta_{i})=x_{i}^{t}(a_{i}^{t},a_{j}^{t})-\beta_{i}\max(x_{i}^{t}(a_{i}^{t},a_{j}^{t})-x_{j}^{t}(a_{i}^{t},a_{j}^{t}),0)$

Trustee: $U_{j}^{t}(a_{i}^{t},a_{j}^{t};\beta_{j})=x_{j}^{t}(a_{i}^{t},a_{j}^{t})-\beta_{j}\max(x_{j}^{t}(a_{i}^{t},a_{j}^{t})-x_{i}^{t}(a_{i}^{t},a_{j}^{t}),0)$

***Q values.*** Here we write the model for how player *i* forms an estimate of optimal play at each round *t* by calculating the values $Q_{i}^{t}$ of their possible actions $a_{i}^{t}$. The actions are the amounts to invest or to return. The $Q$ values are the expected summed utilities over the next two rounds in future. The utility for player *i* depends on the actions of player *j*, which in turn depends on the type of player *j,* and the reasoning that player *j* does about player *i*. Player *i* does not know player *j*’s type, but can learn about it from the history of their interactions, which, up to round *t* is $\mathbf{D}^{\mathbf{t}}=\{(a_{i}^{1},a_{j}^{1}),\cdots,(a_{i}^{t-1},a_{j}^{t-1})\}$. Formally, player *i* maintains beliefs $\mathbf{B}_{\mathbf{i}}^{\mathbf{t}}$, in the form of a probability distribution over the type of player *j*, and computes expected utilities by averaging over these beliefs. Bayes theorem is used to update the beliefs based on evidence.

The $Q_{i}^{t}$ value on round t is a sum of two expectations:

$$Q_{i}^{t}(a_{i}^{t},\mathbf{B}_{\mathbf{i}}^{\mathbf{t}})=\left\langle U_{i}^{t}\right\rangle_{\mathbf{B}_{\mathbf{i}}^{\mathbf{t}}}+\sum_{a_{j}^{t}} P(a_{j}^{t}|\{a_{i}^{t},\mathbf{D}^{\mathbf{t}}\})\sum_{a_{i}^{t+1}} Q_{i}^{t+1}(a_{i}^{t+1},\mathbf{B}_{\mathbf{i}}^{\mathbf{t}+1})P(a_{i}^{t+1}|\{a_{i}^{t},a_{j}^{t},\mathbf{D}^{\mathbf{t}}\})$$

$$(2)$$

The first is the utility of the exchange on that round. This is

$$\left\langle U_{i}^{t}\right\rangle_{\mathbf{B}_{\mathbf{i}}^{\mathbf{t}}}=\sum_{a_{j}^{t}} P(a_{j}^{t}|\{a_{i}^{t},\mathbf{D}^{\mathbf{t}}\})U_{i}(a_{i}^{t},a_{j}^{t};\beta_{i})$$

where, for convenience, we write as a function of the possible actions *a* of player *j* rather than the money this player earns. The second term in the expectation concerns the value of the future two rounds in the exchange (except in the last round, where this term is 0). This is thus an average over values on round *t+1*, where the new beliefs take account of the action $a_{i}^{t}$ being considered by player *i*, and all the possible actions $a_{j}^{t}$ of player *j*. Equation (2) is a form of Bellman equation.

All the beliefs are captured with multinomial distributions, and are estimated by simulating the partner’s play.

***Choosing actions.*** To choose an action, players use a softmax policy based on the values of the state-action pairs. The Softmax action selection rule is a probabilistic way to go from a set of state-action values to an action (as opposed to hard max whereby you would always choose the action with greatest value).

The probability of choosing action $a_{i}^{t}$ given game history $\mathbf{D}^{\mathbf{t}}$ for player *i*  is:

$$P(a_{i}^{t}=a|\mathbf{D}^{\mathbf{t}})=\frac{\exp(\phi Q_{i}^{t}(a_{i}^{t},\mathbf{B}_{\mathbf{i}}^{\mathbf{t}}))}{\sum_{a} \exp(\phi Q_{i}^{t}(a,\mathbf{B}_{\mathbf{i}}^{\mathbf{t}}))}$$

Here is the inverse temperature parameter. It controls how sharp the action selection is. For fixed as $\phi\to0$ the distribution tends to the uniform distribution. We used two temperature settings: and . For each player, we optimized the temperature according to the best log-likelihood resulted from fitting the model to actual behavior.

***Belief representation.*** As described above, a player’s type is defined by his guilt coefficient $\beta\in[0,\text{ }1]$. We discretized *β* and assumed $\beta\in\{0,\text{ }0.25,\text{ }0.5,\text{ }0.75,\text{ }1\}$. A belief about type *β* described a player’s uncertainty over the probability of the five *β* values, i.e. a probability density *ρ* over weights $\lambda=(\lambda_{1},\lambda_{2},\lambda_{3},\lambda_{4},\lambda_{5})\in R^{5},\lambda_{i}\geq0$, and $\sum_{i=1}^{5} \lambda_{i}=1$. The density *ρ* takes the form of the Dirichlet distribution (the conjugate priors for the multinomial distribution):

$$\rho(\lambda;\theta)=\frac{1}{\mathbf{Z}(\theta)}\prod_{i=1}^{5} \lambda_{i}^{\theta_{i}-1}$$

where $\theta=(\theta_{1},\theta_{2},\theta_{3},\theta_{4},\theta_{5})>0$ are the hyperparameters, and the normalizing factor $Z(\theta)$ is a beta function.

To compute the $Q$ values using Eq. 2, a player *і* assesses the probability of his opponent *ј*’s action $a_{j}^{t}$ given current game history, $P(a_{j}^{t}|\{a_{i}^{t},\mathbf{D}^{\mathbf{t}}\})$, which involves computing integrals over the 4-simplex belief space:

$$P(a_{j}^{t}|\{a_{i}^{t},\mathbf{D}^{\mathbf{t}}\})=\int\sum_{l=1}^{5} P(a_{j}^{t}|\beta=\frac{l-1}{4})\lambda_{l}\rho(\lambda;\theta)d\lambda$$

We performed the numerical integration using Gaussian quadrature over the belief space.

***Depth-of-thought and belief update.*** One subtlety that arises is that computing $Q$ requires , through both its explicit appearance in the Bellman equation and through $\mathbf{B}_{\mathbf{i}}^{\mathbf{t}+1}$, *i*’s updated beliefs about types, given that *i* chooses $a_{i}^{t}$ and *j* chooses $a_{j}^{t}$. Consequently, an agent must compute the $Q$ values of their opponent, leading to an infinite regress. To avoid this, we assume that a player reasons at a hierarchical level (as in cognitive hierarchy theory^32^), and assume their opponent reasons at ***one level lower***. Players can have three cognitive levels, or depth-of-thought. Level 0 player *i* simply computes the immediate utility value of player *j*, $U_{j}^{t}$, and uses it to compute $P(a_{j}^{t}|\{a_{i}^{t},\mathbf{D}^{\mathbf{t}}\})$ through the softmax function, and updates their beliefs. A level 1 player assumes that her partner *j* is level 0, and simulates j’s play by computing $Q_{j}^{t}$. Similarly, a level 2 player assumes the partner is level 1, and computes her partner’s $Q$ values to get $P(a_{j}^{t}|\{a_{i}^{t},\mathbf{D}^{\mathbf{t}}\})$. Beliefs are updated as follows:

A level 0 player *i* does not simulate the opponent’s play. It computes the likelihood of observing opponent’s action $a_{j}^{t}$ using the immediate utility for five possible guilt coefficients $\beta^{l}=\frac{l-1}{4},\text{ }l\in[1,\text{ }5]$:

$$P(a_{j}^{t}|\{a_{i}^{t},\mathbf{D}^{\mathbf{t}}\},\beta_{j}=\beta^{l})=\frac{\exp(\phi U_{j}(a_{i}^{t},a_{j}^{t}))}{\sum_{a} \exp(\phi U_{j}(a_{i}^{t},a))}$$

A level 1 or 2 player *i* ($k$= 1 or 2, depth-of-thought) regards the opponent as a level player and simulates her play. It computes the likelihood of observing opponent’s action using the $Q$ value:

$$P(a_{j}^{t}|\{a_{i}^{t},\mathbf{D}^{\mathbf{t}}\},\beta_{j}=\beta^{l})=\frac{\exp(\phi Q_{j}^{t}(a_{j}^{t},\mathbf{B}_{\mathbf{j}}^{\mathbf{t}}))}{\sum_{a} \exp(\phi Q_{j}^{t}(a,\mathbf{B}_{\mathbf{j}}^{\mathbf{t}}))}$$

The belief update follows Bayes rule by updating the hyperparameters *θ* of the Dirichlet distribution. We set the prior belief as: $\theta_{1}=\theta_{2}=\theta_{3}=\theta_{4}=\theta_{5}=1$. The posterior belief is given by a Dirichlet distribution with hyperparameters given by:

$$\theta_{l}^{'}=\theta_{l}+P(a_{j}^{t}|\{a_{i}^{t},\mathbf{D}^{\mathbf{t}}\},\beta_{j}=\beta^{l})$$

***Behavioral classification.*** We then applied the statistical inverse of the generative model above and described in the main text to classify individual players according to their behavior in the sequential trust game.

Since the game is Markovian, we can calculate the likelihood of player *i* taking the action sequence $\{a_{i}^{t},t=1,\ldots,10\}$ given her type , prior beliefs $\mathbf{B}_{\mathbf{i}}^{0}$ and depth-of-thought $k_{i}$ as:

$$P(\{a_{i}^{t}\}|\{t\})=P(a_{i}^{1}|\mathbf{B}_{\mathbf{i}}^{\mathbf{0}},\beta_{i},k_{i})\prod_{t==2}^{10} P(a_{i}^{t}|\mathbf{D}^{\mathbf{t}},\beta_{i},k_{i})$$

where $P(a_{i}^{1}|\mathbf{B}_{\mathbf{i}}^{\mathbf{0}},\beta_{i},k_{i})$ is the probability of initial action $a_{i}^{1}$ given by the softmax distribution, prior beliefs $\mathbf{B}_{\mathbf{i}}^{0}$ and depth-of-thought $k_{i}$, and $P(a_{i}^{t}|\mathbf{D}^{\mathbf{t}},\beta_{i},k_{i})$ is the probability of taking action $a_{i}^{t}$ after updating beliefs $\mathbf{B}_{\mathbf{i}}^{t}$ from previous beliefs $\mathbf{B}_{\mathbf{i}}^{t-1}$ upon observing the history of moves $\mathbf{D}^{\mathbf{t}}$.

Finally, we classify the players for their type and depth-of-thought $k$ by finding values of that maximize $\log P(\mathbf{D}^{\mathbf{10}}|\beta_{i},\mathbf{B}_{\mathbf{i}}^{\mathbf{0}},k_{i})$.

**Reinforcement learning model**

For comparison with a perhaps conceptually simpler model, we constructed a reinforcement learning model. As in the computational theory-of-mind model, we used the Fehr-Schmidt utility function and only considered the guilt coefficient. So a player’s immediate utility or reward was given by

$$U_{i}(x_{i},x_{j};\beta_{i})=x_{i}-\beta_{i}\max(x_{i}-x_{j},0)\text{ }$$

where $x_{i}$ is the player’s immediate payout, and $x_{j}$ is the other player’s immediate payout.

We considered five possible actions for investors and trustees. Investor’s actions$a_{i}\in\{0,\text{ }5,\text{ }10,\text{ }15,\text{ }20\}$, and Trustee’s actions $a_{j}\in\{0,\text{ }\frac{3}{4}a_{i},\text{ }\frac{3}{2}a_{i}\text{, }\frac{9}{4}a_{i},\text{ }3a_{i}\}$. In this reinforcement learning model, players learned a value associate with each action. For improved generalization, since the number of rounds was limited, players learned a linear function for the action-values:

$$Q(a)=ka+b$$

At round *t*, player $i$ computed a prediction error $\delta_{t}$ after observing the opponent $j$’s action:

$\delta_{t}=U_{i}^{t}(a_{i}^{t},a_{j}^{t},\beta_{i})-Q(a_{i}^{t})$.

After calculating the prediction error, the value was then updated according to the temporal difference rule: $Q_{new}(a_{i}^{t})=Q_{old}(a_{i}^{t})+\varepsilon\delta_{t}$, where *ε* was the learning rate. The best parameters $\theta=(k,b)$ for the linear fit were then recalculated using the new value of the chosen action and the old values of the unchosen actions to get . The values for all the actions were then updated with the new parameters $\hat{\theta}$.

To fit this model to the actual behavior, we computed the probability of selecting action $a_{i}^{t}$ using a softmax likelihood, given the action-value $Q(a_{i}^{t})$:

$$P(a_{i}^{t})=\frac{\exp(\phi Q(a_{i}^{t}))}{\sum_{a} \exp(\phi Q(a))},\text{ }\phi=1$$

The negative log-likelihood of the game history *D* given the parameters $k,b,\beta_{i},\beta_{j}$ was given by:

$$L(D|k,b,\beta_{i},\beta_{j})=\sum_{t=1}^{10} -\frac{1}{2}(\log P(a_{i}^{t})+\log P(a_{j}^{t}))$$

The values of *k* ranged between 0 and 30, and the values of *b* ranged between 0 and 20. The learning rate $\varepsilon=\{0,\text{ 0.25, }0.5,\text{ 1\}}$. For each subject pair, we calculated the negative log-likelihood, and chose the best fit values of $\beta_{i}$ and $\beta_{j}$. We then took the averaged negative log-likelihoods over all subject pairs within a group (we had four groups, Impersonal, Personal, BPD and BPD controls).

We report the best fitting parameters *k* and *b* and the averaged negative log-likelihood for different learning rates in Table S1 and Table S2, respectively. We found that the parameters maximizing the log-likelihood were $k=0$, and $\varepsilon=0$. Note that if all the five actions were chosen with equal probability, the negative log-likelihood would take the value $-10\cdot\log(\frac{1}{5})=16.09$. Thus the model degenerated to the case where the values were uniform, no learning occurred, and all actions were selected equally. Table S2 also includes the negative log-likelihood for the computational theory-of-mind model. Comparison demonstrates that the computational theory-of-mind model provides a better fit.

**References**

1. Kaelbling LP, Littman ML, Cassandra AR (1998) Planning and acting in partially observable stochastic domains. Artificial Intelligence 101:99-134.
2. Gmytrasiewicz PJ, Doshi PA (2005) Framework for Sequential Planning in Multiagent Settings. JAIR 24:49-79.
